# The extended effect of adsorbed damage-associated molecular patterns and Toll-like receptor 2 signaling on macrophage-material interactions

**DOI:** 10.3389/fbioe.2022.959512

**Published:** 2022-08-26

**Authors:** Anuj Kaushal, Yuxi Zhang, Laurel L. Ballantyne, Lindsay E. Fitzpatrick

**Affiliations:** ^1^ Department of Chemical Engineering, Queen’s University, Kingston, ON, Canada; ^2^ The Centre for Health Innovation, Queen’s University and the Kingston Health Sciences Centre, Kingston, ON, Canada

**Keywords:** host response, biomaterial, macrophage, damage-associate molecular patterns (DAMPs), Toll-like receptor 2, inflammation, Teflon AF 1600, TGF–β1

## Abstract

Implanted biomaterials elicit an immune-mediated foreign body reaction (FBR) that results in the fibrous encapsulation of the implant and can critically impact the performance of some implants. Consequently, understanding the molecular mechanisms that underpin cell-materials interactions that initiate biomaterial-induced inflammation and fibrosis is critical to improving the performance of biomaterial implants negatively impacted by the FBR. Damage-associated molecular patterns (DAMPs) are endogenous mediators of inflammation that are released upon tissue injury and induce sterile inflammation *via* Toll-like receptors (TLRs). However, the prevalence of DAMPs within the adsorbed protein layer on material surfaces and their role mediating cell-material interactions is unclear. Previously, our group demonstrated that molecules in fibroblast lysates adsorbed to various biomaterials and induced a potent TLR2-dependent inflammatory response in macrophages at 24 h. In this study, we examined the extended response of RAW-Blue reporter macrophages on lysate or serum-adsorbed Teflon™ AF surfaces to understand the potential role of adsorbed DAMPs in macrophage-material interactions at later time points. Lysate-conditioned surfaces maintained increased nuclear factor kappa B (NF-κB) and activator protein 1 (AP-1) transcription factor activity and increased expression Regulated upon Activation, Normal T Cell Expressed and Presumably Secreted (RANTES/CCL5) at 72 h and 120 h, compared to FBS-conditioned surfaces. In contrast, monocyte chemoattractant protein 1 (MCP-1/CCL2) was only elevated at 72 h in lysate conditions. Transforming growth factor beta 1 (TGF-β1) secretion was significantly increased on lysate-conditioned surfaces, while conditioned media from macrophages on lysate-conditioned surfaces induced alpha smooth muscle actin (αSMA) expression in 3T3 fibroblasts. TLR2 neutralizing antibody treatment significantly decreased NF-κB/AP-1 activity and attenuated TGF-β1 expression at both time points, and MCP-1 and RANTES at 72 h. Finally, multinucleated cells were observed on lysate-conditioned surfaces at 72 h, indicating adsorbed DAMPs induced a fusion permissive environment for adherent macrophages. This study demonstrates that adsorbed DAMPs continue to influence macrophage-material responses beyond the initial 24-h period and maintain a pro-inflammatory and fibrotic response that models aspects of the early FBR. Furthermore, the transient inhibition of TLR2 continued to exert an effect at these later time points, suggesting TLR2 may be a target for therapeutic interventions in FBR.

## 1 Introduction

The foreign body reaction (FBR) is an immune-mediated response to implanted biomaterials that often results in chronic inflammation and fibrous encapsulation of the implant. While the severity and impact of the FBR varies based on the implant properties and location, degree of tissue trauma and individual patient factors, the FBR can compromise device performance, and may even cause device failure and necessitate the surgical revision or removal (Anderson, 1988, 2015; Anderson et al., 2008; Noskovicova et al., 2021). Our understanding of the nuanced cascade of events, the diversity and plasticity of relevant cell populations and the influence of material properties involved of the progression of the FBR has continued to evolve. Yet, recent advances on understanding the dynamic signaling of innate and adaptive immune cells and their crosstalk with stromal cells within implant sites has revealed that many aspects of the FBR remain unclear and merit further investigation (Sadtler et al., 2019; Wolf et al., 2019; Moore et al., 2021; Fernandez-Yague et al., 2022). As such, the FBR remains a significant challenge for many biomedical devices used in clinical applications, including the formation of foreign body granulomas within surgical meshes and tissue fillers that cause adhesions and pain, fibrous capsular contractures surrounding silicone breast implants and issues with reliability, noise and the constant need for recalibration for implantable glucose sensors (Rigla et al., 2018; Singh et al., 2018; Lemperle et al., 2020; Kuehlmann et al., 2021; Klinge et al., 2022). Furthermore, biomaterial-associated inflammation and fibrous encapsulation also continues to pose a challenge in the development of advanced biomedical technologies that require an unimpeded interface with the surrounding tissue to facilitate biosensing or the transport of drugs, nutrients or other molecules to and/or from the implant surface (Veiseh and Vegas, 2019; Noskovicova et al., 2021). For example, the success of intraneural interfaces is currently limited by inefficient signal transduction and increased electrical impedance that develops with fibrotic tissue deposition at the neural interface (Lotti et al., 2017; Carnicer-Lombarte et al., 2021), while cell encapsulation devices experience mass transport limitations due to the fibrous capsule formation and lack of vascularization at the tissue/material interface (U.S. National Library of Medicine, 2022).

Macro-scaled (i.e., non-phagocytosable), synthetic biomaterials generally elicit a classic FBR, which can be divided into two phases. The early inflammatory response to an implanted biomaterials consists of tissue injury/hemostasis, protein adsorption on the material surface and an acute inflammatory response mediated by neutrophils and mast cells during the first 2 days, then followed by macrophages (Martin and Garcia, 2021). During this early phase, macrophages recruited to the implant site display a predominantly pro-inflammatory or M1-like phenotype and secrete mainly proinflammatory cytokines, including interleukin 1β (IL-1β), IL-6, monocyte chemoattractant protein 1 (MCP-1/CCL2) and tumor necrosis factor alpha (TNF-α) (Shireman et al., 2006; Anderson et al., 2008; Wynn et al., 2013; de la Oliva et al., 2018). Due to the presence of the biomaterial, acute inflammation fails to resolve and instead transitions into chronic inflammation and FBR, characterized by an increased presence of macrophages at the implant surface and the fusion of adherent macrophages to form multinucleated foreign body giant cells (FBGC) (Anderson et al., 2008; Martin and Garcia, 2021). Adherent macrophages and FBGC attempt to degrade the implant by releasing degradative enzymes and reactive oxygen species, and also release cytokines that activate fibroblasts from the surrounding tissue to secrete a collagen-rich extracellular matrix (ECM), eventually forming a fibrous capsule around the implant (Anderson et al., 2008). The transition from the acute to chronic inflammation is accompanied by a shift in the cytokine profile to include cytokines generally associated with late/sustained inflammation, such as MCP-1, and Regulated upon Activation, Normal T Cell Expressed and Presumably Secreted (RANTES/CCL5), and with tissue regeneration and fibrosis, including transforming growth factor beta (TGF-β), platelet-derived growth factor (PDGF) and IL-10 (Anderson et al., 2008; Melton et al., 2015; Zhao et al., 2016; Liu and Segura, 2020; Martin and Garcia, 2021). Macrophages and their cross-talk with fibroblasts are well established as central actors in the progression and severity of the FBR, while more recent studies have enriched our understanding of the roles of adaptive immune cells, including T cells and B cells, in the complex immune microenvironment surrounding a biomaterial implant during the later stages of chronic inflammation and peri-implant fibrosis (Anderson et al., 2008; Anderson & McNally, 2011; Witherel et al., 2019; Moore et al., 2021).

It is well accepted that the release of damage-associated molecular patterns (DAMPs) by injured cells occurs for any wounding event, without or without the presence of implanted biomaterials (Zhang et al., 2010; Venereau et al., 2015; Roh and Sohn, 2018). Whereas DAMPs within a normal wound are cleared as inflammation resolves and the normal wound healing response progresses, we and others have hypothesized that the interaction between released DAMPs and the implant surface contributes to the activation of biomaterial-adherent macrophages and the propagation of the chronic inflammation and fibrosis typically observed in a FBR (Rogers and Babensee, 2010; McKiel and Fitzpatrick, 2018; Amer et al., 2019). The initial event in biomaterial host responses is the blood-material interaction that occurs immediately upon insertion of the implant, in which blood-derived proteins and non-proteinaceous molecules adsorb to the surface of the material (Swartzlander et al., 2015). This adsorbed protein layer is thought to mediate downstream cell-material interactions, and the composition and conformation of proteins within this layer is influenced, to a degree, by material properties, including chemical composition and surface charge, roughness and polymer chain flexibility (Vyner et al., 2013; Vyner & Amsden, 2016; Wells et al., 2017). However, the contribution of tissue-derived DAMPs within the adsorbed protein layer and their potential impact on the progression of the FBR has received little attention, despite the critical role DAMPs and their receptors, such as Toll-like receptors (TLR), play in initiating sterile inflammation *via* activation of pro-inflammatory nuclear factor kappa B (NF-κB) and activator protein 1 (AP-1) transcription factors (Zhang et al., 2010; Roh and Sohn, 2018).

Previously, our group studied the ability of cell-derived DAMPs within fibroblast lysate to adsorb on various hydrophobic biomaterial surfaces and induce a pro-inflammatory response from a mouse macrophage cell line, even when DAMP-containing lysates were diluted ten-fold in serum or plasma prior to adsorption (McKiel and Fitzpatrick, 2018; McKiel et al., 2020). Macrophage activation on lysate-conditioned polymethylmethacrylate, polydimethylsiloxane and amorphous fluoropolymer (Teflon™ AF 1600) surfaces was found be dependent primarily on TLR2, with TLR4 playing a more modest role (McKiel and Fitzpatrick, 2018; McKiel et al., 2020). However, as these studies only focused on the macrophage response at 24 h, it is unclear if the adsorbed DAMPs would continue to have a significant effect on the macrophage-material interactions beyond this acute time frame.

The current study aimed to characterize the extended effect of adsorbed DAMPs on NF-κB and AP-1 transcription factor activity and the expression of pro-inflammatory and pro-fibrotic cytokines at 72 hand 120 h *in vitro*. Indirect co-cultures with mouse fibroblasts using macrophage conditioned media were used to examine the profibrotic macrophage response to DAMP-adsorbed surfaces, compared to serum-adsorbed surfaces. Finally, we also explored how the acute inhibition of TLR2 by a neutralizing antibody influenced the macrophage-material response at these later time points to gain insights into the role of TLR2 beyond the acute initiation phase of inflammation.

Amorphous fluoropolymer Teflon™ AF was selected as the material substrate in the present study to model the polytetrafluoroethylene (PTFE) cannulas of insulin infusion sets (IIS), which are used with insulin pumps for continuous subcutaneous insulin infusion (CSII). CSII is a treatment option for managing Type 1 Diabetes, in which insulin analogues are delivered to the subcutaneous tissue (Schmid et al., 2010). A major limitation of CSII is the short wear times and the poor reliability of IIS, which can lead to the development of unexplained hyperglycemia and diabetic ketoacidosis (Hauzenberger et al., 2017; Hauzenberger et al., 2018). While the underlying cause of unexplained hyperglycemia is unclear, the tissue response to the IIS cannulas is thought to contribute to variable insulin adsorption at the infusion site (Schmid et al., 2010). Therefore, understanding the interaction between adsorbed DAMPs and TLR2 signaling in macrophages on model Teflon™ AF surfaces will contribute to better understanding the host response to PTFE cannulas.

## 2 Materials and methods

### 2.1 Cell lines

RAW-Blue™ (Invivogen, San Diego, CA, United States; male) macrophages are NF-κB/AP-1 reporter cells that stably express secreted embryonic alkaline phosphatase (SEAP) under the control of NF-κB and AP-1 transcription factors. RAW-Blue™ macrophages were maintained in Dulbecco’s Modified Eagle’s Medium (DMEM, Sigma-Aldrich, Oakville, ON, Canada) supplemented with 10% fetal bovine serum (FBS, Wisent, St. Bruno, QC, Canada), 5 μg/ml Plasmocin (Invivogen), and 200 μg/ml Zeocin (Invivogen). The RAW 264.7 (ATCC, Manassas, VA, United States; male) murine macrophage cell line was cultured in DMEM supplemented with 10% FBS and 1% Penicillin/Streptomycin (Sigma-Aldrich). NIH3T3 (ATCC) murine fibroblasts were cultured in DMEM supplemented to contain 10% FBS and 1% Penicillin/Streptomycin. All cell lines were maintained in their respective complete growth media and sub-cultured when cultures reach approximately 70% confluence. RAW-Blue™ and RAW 264.7 cultures were used to up passage 15, and NIH/3T3 fibroblast cultures were used up to passage 10.

### 2.2 Lysate preparation

3T3 fibroblasts were used to generate DAMP-containing lysate, as previously described (McKiel and Fitzpatrick, 2018; McKiel et al., 2020). To generate the lysate, the fibroblasts were detached using TrypLE (Fischer Scientific, Ottawa, ON, Canada) and were counted using a hemocytometer after staining with Trypan Blue (Fischer Scientific). The cells were then centrifuged at 200 xg for 5 min, resuspended at 5 × 10^6^ cells/ml, and freeze-thaw cycled three times using a −80°C freezer and a 37°C water bath. The protein concentration of the lysate was quantified using a microBCA assay (Fischer Scientific) according to the manufacturer’s instructions. The lysate was then diluted to 625 μg/ml in sterile PBS, aliquoted and stored in a −80°C freezer until use.

### 2.3 Teflon™ AF 1,600 coatings

In this study, an amorphous fluoropolymer, Teflon™ AF 1,600 (Sigma-Aldrich) was used as an *in vitro* cell culture substrate to model the surface of polytetrafluoroethylene (PTFE) insulin infusion cannulas. Teflon™ AF 1,600 is an amorphous copolymer of 65 mol% 2-bistrifluoromethyl-4,5-difluoro-1,3-dioxole (PDD) and 35 mol% tetrafluoroethylene (TFE) and shares many characteristics with semi-crystalline PTFE, including wettability (Anamelechi et al., 2005). However, the surface chemistry of Teflon™ AF 1600 does differ from PTFE due to the oxygen content of the PDD comonomer (Anamelechi et al., 2005). Teflon™ AF 1,600 is also soluble in perfluorinated solvents, which allows films to be cast from solutions. Furthermore, Teflon™ AF 1,600 has excellent optical clarity due to its amorphous structure, which beneficial for cell culture studies that rely of visualization of adherent cells on the fluoropolymer surfaces.

Teflon™ AF 1,600, hereafter referred to simply as Teflon™ AF, was dissolved in a fluorinated solvent (FC-40; Sigma-Aldrich) to create a 1 mg/ml solution and used to coat 24 well non-tissue culture treated polystyrene plates, as previously described (Godek et al., 2009; McKiel et al., 2020). Briefly, 500 µl of 1 mg/ml Teflon™ AF solution was added to each well, the well plates were placed in a vacuum oven at 22.5 inHg and 40°C for 72 h to remove the fluorinated solvent. The wells were then decontaminated with 70% ethanol and subsequently washed three times with endotoxin free water (McKiel and Fitzpatrick, 2018). Prior to use, Teflon™ AF-coated plates were placed under UV light for 30 min.

Each plate was also tested for endotoxin using a LAL Pyrochrome kit, according to the manufacturer’s provided instructions (CapeCod and Associates, East Falmouth, MA). Three wells from each plate were tested in duplicate, as described previously (McKiel and Fitzpatrick, 2018), using 100 µl of endotoxin free water per well. All endotoxin levels were below the lower limit of the assay (0.05 EU/ml).

### 2.4 Macrophage cultures on lysate or 10% FBS preconditioned Teflon™ AF surfaces

Teflon™ AF-coated wells were pre-conditioned with either 3T3 lysate (125 μg/ml) or 10% FBS (1,600 μg/ml) for 1 h at room temperature, then washed with sterile PBS three times for 5 min each time. RAW-Blue™ cells were seeded in Teflon™ AF-coated wells at 1 × 10^4^ cells/well (5 × 10^3^ cells/cm^2^) in DMEM supplemented with 10% heat-inactivated fetal bovine serum (HI-FBS), 5 μg/ml Plasmocin and 200 μg/ml Zeocin, while RAW 264.7 macrophages were seeded in their normal growth media. The use of heat-inactivated serum is required to eliminate the activity of any serum alkaline phosphatase in the SEAP-dependent NF-κB/AP-1 reporter assay to avoid false positive results. As a positive control for TLR2-induced macrophages activation, Pam3CSK4 (150 ng/ml; Invivogen) was added to a RAW-Blue™ macrophages seeded in Teflon™ AF-coated wells with adsorbed-serum. To block TLR2 signaling, RAW-Blue™ or RAW 264.7 macrophages were incubated in 2.5 μg/ml of neutralizing TLR2 antibody, anti-mTLR2-IgG (cat. no. mabg-mtlr2, Invivogen) for 1 h prior to seeding on the Teflon™ AF surfaces.

Plates were then incubated at 37°C and 5% CO_2_ for 120 h. Partial media changes (50% of total volume) were performed at 48 h and 96 h. At 72 h and 120 h, supernatant samples were collected and either used immediately in a QUANTI-Blue assay (Invivogen) or stored at −80° to be used for cytokine analysis.

### 2.5 QUANTI-Blue NF-κB/AP-1 reporter assay

The NF-κB/AP-1 activity in macrophages was measured indirectly as SEAP activity using a colorimetric enzymatic assay for alkaline phosphatase activity (QUANTI-Blue, Invivogen). Supernatant samples from RAW-Blue™ macrophages were diluted in QUANTI-Blue reagent in 96 well plates and incubated for 2.5 h, according to the manufacturer’s instructions. The absorbance was measured in duplicate at 620 nm using a Biotek Synergy H1 microplate reader. All absorbance values were normalized to the absorbance of the negative assay control (media with no cells).

### 2.6 QuantiFluor assay

The number of cells present after 120 h of incubation were measured using the Promega QuantiFluor dsDNA System (Fischer Scientific). Supernatant was removed from each well and cells were washed twice with PBS before being placed in the −80°C freezer. A standard curve ranging from 1,000 to 200,000 cells was created using RAW-Blue™ cells, which were counted on a hemocytometer, diluted in PBS, and pelleted in triplicate in a well plate before being placed in a −80°C freezer. A 4x QuantiFluor dsDNA dye dilution was prepared by diluting in 1x TE buffer (supplied in kit). The dilution was selected based on an expected cell count of 200,000 cells per well. Cells were then removed from the freezer and 440 µl of 4x QuantiFluor dsDNA dye was added to each well of frozen cells to completely lyse the cells. The samples were then transferred to a 96 well plate and incubated for 5 min at room temperature, protected from light. Fluorescence was measured at 480 nm excitation maximum and 520 nm emission maximum using a Biotek Synergy H1 microplate reader.

### 2.7 Cytokine immunoassays

The concentration of cytokines in the supernatant of RAW-Blue™ macrophage cultures on Teflon™ AF surfaces was measured using immunoassays. A Milliplex Luminex assay (Millipore Sigma-Aldrich Canada, Oakville, ON, Canada) measured the concentrations of IL-1β, IL-6, IL-10, MCP-1, and RANTES in undiluted supernatant samples, following the manufacturer’s instructions. Each sample was measured in duplicate, and concentrations were interpolated from standard curves, which ranged from 10,000 pg/ml to 3.2 pg/ml for each cytokine. Out of range (OOR) data within the Luminex panels, which accounted for less than 10% of samples, were discarded from the analysis and treated as missing values.

The supernatant concentration of TGF-β1 was measured using an ELISA kit (Fischer Scientific), according to the manufacturer’s instructions. The samples were diluted 1:5 in assay buffer and were measured in duplicate. The absorbance values were read at 450 nm and 620 nm using a Biotek Synergy H1 microplate reader. Concentrations of the diluted samples were interpolated from the standard curve ranging from 2000 pg/ml to 31.25 pg/ml. ELISA cytokine measurements that could not be interpolated (i.e., absorbances below the range of the standard curve) were treated as missing values.

### 2.8 Foreign body giant cell quantification by immunofluorescence microscopy

Lysate (125 μg/cm^2^) or 10% FBS (∼1,600 μg/cm^2^) were used to pre-condition the surfaces of TCPS 96 well plates for 60 min, then seeded with RAW-Blue reporter macrophages (1.5 × 10^5^ cells/cm^2^; DMEM supplemented with 10% FBS, 5 μg/ml plasmocin and 200 μg/ml zeocin) and incubated for 72 h at 37°C and 5% CO_2_.

After 72 h, cell morphology and quantification of fusion and multinucleated cell size was assessed using brightfield and fluorescence microscopy. The Raw-Blue™ macrophages were incubated for 20 min at 37°C with staining solution containing 2 drops/ml Hoechst 33342 (NucBlue, ThermoFisher Scientific; Ex/Em: 360/460 nm) and 10 μg/ml Wheat Germ Agglutinin (WGA) Alexa Fluor™ 488 conjugate (ThermoFisher Scientific; Ex/Em: 495/519 nm) to demarcate the nucleus and plasma membrane, respectively. Labeled macrophages were imaged at 40x using a Nikon Eclipse Ti2 microscope, using the NIS Elements Advanced Research imaging software. Three fields of view were randomly selected near the center area of each well. Nuclei enumeration (Hoechst) and size measurements of multinucleated cells (WGA-labeled area) was performed in ImageJ (Schneider et al., 2012). FBGC were classified as cells containing three or more nuclei to exclude cells undergoing mitosis (Saleh et al., 2020). The percent fusion was quantified as

$$\text{\% fusion}=\frac{\text{number of nuclei in FBGCs}}{\text{total number of nuclei}}\times \;100\text{\%}$$ (1)

### 2.9 Fibroblasts cultured in macrophage conditioned media

#### 2.9.1 Macrophage conditioned media

Teflon™ AF coated 24 well plates were incubated with lysate and 10% FBS as described above. RAW 264.7 cells were seeded in the wells at a concentration of 1 × 10^4^ cells/well (5 × 10^3^ cells/cm^2^) and incubated at 37°C and 5% CO_2_ for 120 h. Media changes (50%) were performed at 48 h and 96 h. At 72 h and 120 h, the supernatant was transferred to a microcentrifuge tube and centrifuged at 1,000xg for 10 min at 4°C. The supernatant was then transferred to a new microcentrifuge tube and stored at −80°C. RAW 264.7 cells grown in media containing Pam3CSK4 (150 ng/ml) were used as a positive control for TLR2 signaling. For TLR2 inhibition, RAW 264.7 macrophages were incubated with a neutralizing TLR2 antibody, anti-mTLR2-IgG (2.5 μg/ml), for 1 h prior to seeding on Teflon™ AF surfaces.

#### 2.9.2 Fibroblast culture in macrophage conditioned media

3T3 cells were seeded into 24 well TCPS plates and 8 well µ-slides (Ibidi, Madison, WI, United States) at a concentration of 4 × 10^3^ cells/cm^2^. Conditioned media from RAW 264.7 cells was added to the wells such that the conditioned media made up 30% of the total media. 3T3 cells treated with myofibroblast differentiation inducer, TGF-β1 (10 ng/ml), were used as a positive control and 3T3 cells grown with no conditioned media were used as a negative control. Fibroblasts were incubated at 37°C and 5% CO_2_ for 48 h. The 24 well TCPS plates were used to isolate RNA for qPCR, while the 8 well µ-slides were used for fluorescence microscopy.

#### 2.9.3 Fluorescence microscopy

All staining procedures were performed in the dark. The supernatant was removed from the 8 well µ-slides and each well was washed with PBS for 5 min. The fibroblasts were fixed using 4% paraformaldehyde (Sigma-Aldrich) for 15 min, then washed twice with PBS. The cells were permeabilized with 0.5% Triton-X (Fischer Scientific) for 5 min, then washed with PBS. A FITC-conjugated α-smooth muscle actin (αSMA) monoclonal antibody (Sigma-Aldrich) was diluted to 1:250 in a 1% bovine serum albumin (BSA; Sigma-Aldrich) in PBS solution and added to each well for 20 h at 4°C. The wells were rinsed twice with PBS. AlexaFluor 555 conjugated phalloidin (Fischer Scientific) was diluted to 1:400 in a 1% BSA in PBS solution and added to each well for 90 min at room temperature. The cells were washed twice with PBS and a drop of NucBlue (Fischer Scientific) was added to each well.

Cells were imaged with a Nikon Eclipse Ti2 microscope, using the NIS Elements Advanced Research imaging software. Three representative images of each well were captured at 20x and 40x magnification. All image settings were kept constant between the different conditions. The 40X images were analyzed using ImageJ (Schneider et al., 2012) to determine the fluorescence intensity of each cell in the FITC channel and to determine the size of each cell present in a condition (all channels). Images were opened in ImageJ and the scale was then set using the scale bar in each image. The images were then converted to 16-bit greyscale images and, using the threshold function, the individual cells were highlighted. The measure function was then used to determine the cell area and the average grey scale intensity (fluorescence intensity) of each cell.

#### 2.9.4 Gene expression analysis

Total RNA was extracted from 3T3 fibroblasts using a RNeasy micro kit (Qiagen, Toronto, ON, Canada) with DNase treatment, according to the manufacturer’s instructions. Purified RNA was eluted in RNase-free water and stored at −80°C. RNA concentrations and purity were measured using a Nanodrop One Spectrophotometer (ThermoFisher). All samples had an A260/A280 ratio above 1.8. cDNA was synthesized using iScript™ Reverse Transcription Supermix (Bio-Rad Laboratories, Mississauga, Canada) using 1 µg of RNA in each 20 µl reaction, according to the manufacturer’s instructions.

Custom primers for all genes were designed using PrimerBlast, according to the following criteria: 100–225 base pairs in product length, primer length close to 20 base pairs, primer melting temperature between 57°C and 63°C, a GC content between 40% and 60%, low self-complementarity and low self 3′ complementarity, and unique targets. All primers were validated prior to use for gene expression analysis. A standard dilution curve was obtained from the cDNA with the primers to ensure a linear working range. All primers had an efficiency between 90% and 110%. A melt curve optimization experiment was performed for each gene with annealing temperatures ranging from 54°C—64°C. An annealing temperature of 58°C was chosen for the qPCR protocol. The stability of reference genes Rplp0 and B2m was assessed using GeNorm in qbase+ (Biogazelle), and both reference genes yielded M values below 0.1. Primer sequences and their amplification efficiencies are listed in Table 1. qPCR was performed using SsoAdvanced Universal SYBR Green Supermix (Bio-Rad), in a total reaction volume of 10 µl with 300 nM of forward and reverse primers and 10 ng cDNA in a 384 well plate. Three biological replicates were used for each condition and each sample was plated in triplicate. No template controls (NTC) were included in all assays. The plates were run in a Bio-Rad CFX384 system using the thermal cycling protocol provided in Table 2.

**TABLE 1:** Primer sequences used in the qPCR assay and their amplification efficiencies.

Gene abbreviation	Primer sequences (5′-3′)	GenBank accession number	Efficiency (%)
αSMA	F: GTC​CCA​GAC​ATC​AGG​GAG​TAA	NM_007392.3	102.1
	R: TCG​GAT​ACT​TCA​GCG​TCA​GGA		
OPN	F: GTG​AGA​TTC​GTC​AGA​TTC​ATC​CG	NM_001204201.1	95.7
	R: AGC​AAG​AAA​CTC​TTC​CAA​GCA​A		
Rplp0	F: GGG​CAT​CAC​CAC​GAA​AAT​CTC	NM_007475.5	97.2
	R: CTG​CCG​TTG​TCA​AAC​ACC​T		
B2M	F: TTC​TGG​TGC​TTG​TCT​CAC​TGA	NM_009735.3	95.2
	R: CAG​TAT​GTT​CGG​CTT​CCC​ATT		

**TABLE 2:** Thermal cycling protocol used for qPCR.

Step name	Temperature (oC)	Duration	Number of cycles
1 Polymerase activation	95^o^	30 s	1
2 Denaturation	95^o^C	10 s	40 (steps 2–4)
3 Annealing/extension	58^o^C	30 s	
4 Plate read			
5 Melt curve	65–95^o^C (0.5^o^C increments)	5 s/step	1

Quantification cycles (Cq) were determined using Bio-Rad’s Maestro Software. The relative quantities (RQ) of each gene were determined using Eq. 2, where the efficiency (E) for each gene assumed to be 100% (E = 2). The normalized relative quantity (NRQ) for the genes of interest was then calculated by normalizing the RQ of the genes of interest (GOI) to the geometric mean RQ of the reference (REF) genes (Eq. 3). The relative expression ratio (R) was determined by comparing the NRQ of the treated groups (NRQ_TEST_) with the NRQ of the negative control (NRQ_CONTROL_; Eq. 4). An ANOVA of the log transformed NRQ values (log_2_NRQ) was performed to determine statistical difference among groups using α = 0.05. Changes in gene expression were only considered significant if R was less than 0.67 or greater than 1.5 (0.67 < R < 1.5) and the log_2_NRQ p value was less than 0.05. Error bars were presented as the standard error of the ratio (SE_RATIO_), which was calculated using Eq. 5, as previously described by Rieu and Powers^77^.

$$RQ=1/E^{Cq}\;$$ (2)

$$NRQ=\frac{RQ_{GOI}}{{\left(RQ_{REF1}\times RQ_{REF2}\right)}^{1/2}}$$ (3)

$$R=NRQ_{TEST}/NRQ_{CONTROL}$$ (4)

$$SE_{\mathrm{RATIO}}={\left[\frac{\mathrm{mean NQ}R_{\mathrm{TEST}}}{\mathrm{mean NQ}R_{\mathrm{CONTROL}}}\times \left[\frac{SE_{Test}^{2}}{{\left(\mathrm{mean NQ}R_{\mathrm{TEST}}\right)}^{2}}+\frac{SE_{\mathrm{CONTROL}}^{2}}{{\left(\mathrm{mean NQ}R_{\mathrm{CONTROL}}\right)}^{2}}\right]\right]}^{\frac{1}{2}}$$ (5)

### 2.10 Statistics

Unless otherwise stated, all data was expressed as mean ± standard deviation. Measurements outside of three standard deviations from the mean were considered outliers and excluded from analysis. Data sets with normal distributions (D’Agostino-Pearson test) and equal variances (Brown-Forsythe test) were analyzed using an ordinary one-way ANOVA test with Tukey’s post-hoc test for comparison between all groups. Data sets with normal distributions and unequal variances were analyzed using a Brown-Forsythe and Welch ANOVA test with a Dunnet T3 post-hoc test for multiple comparisons between all groups. For the TGF-β1 ELISA data set, groups with fewer than three detectable concentrations were excluded from statistical analysis, and a unpair two-tailed T-test was perform for the Lysate and Pam3SCK4 groups at each time point. All statistics were performed in Graphpad Prism 9.2.0 with an α value of 0.05 to determine statistical significance. Each experiment was performed three times, with each condition run in triplicate (*n* = 9). For clarity in the figures, only the following ANOVA post-hoc comparisons are shown: 10% FBS (negative control) vs. all conditions, 10% FBS vs. 10% FBS + Inhibitor, Lysate vs. Lysate + Inhibitor, Pam3SCK4 vs. Pam3SCK4+Inhibitor, and Lysate vs. Pam3CSK4.

## 3 Results

### 3.1 TLR2-dependent NF-κB/AP-1 activity in macrophages cultured on lysate-conditioned Teflon™ AF surfaces

**FIGURE 1:**
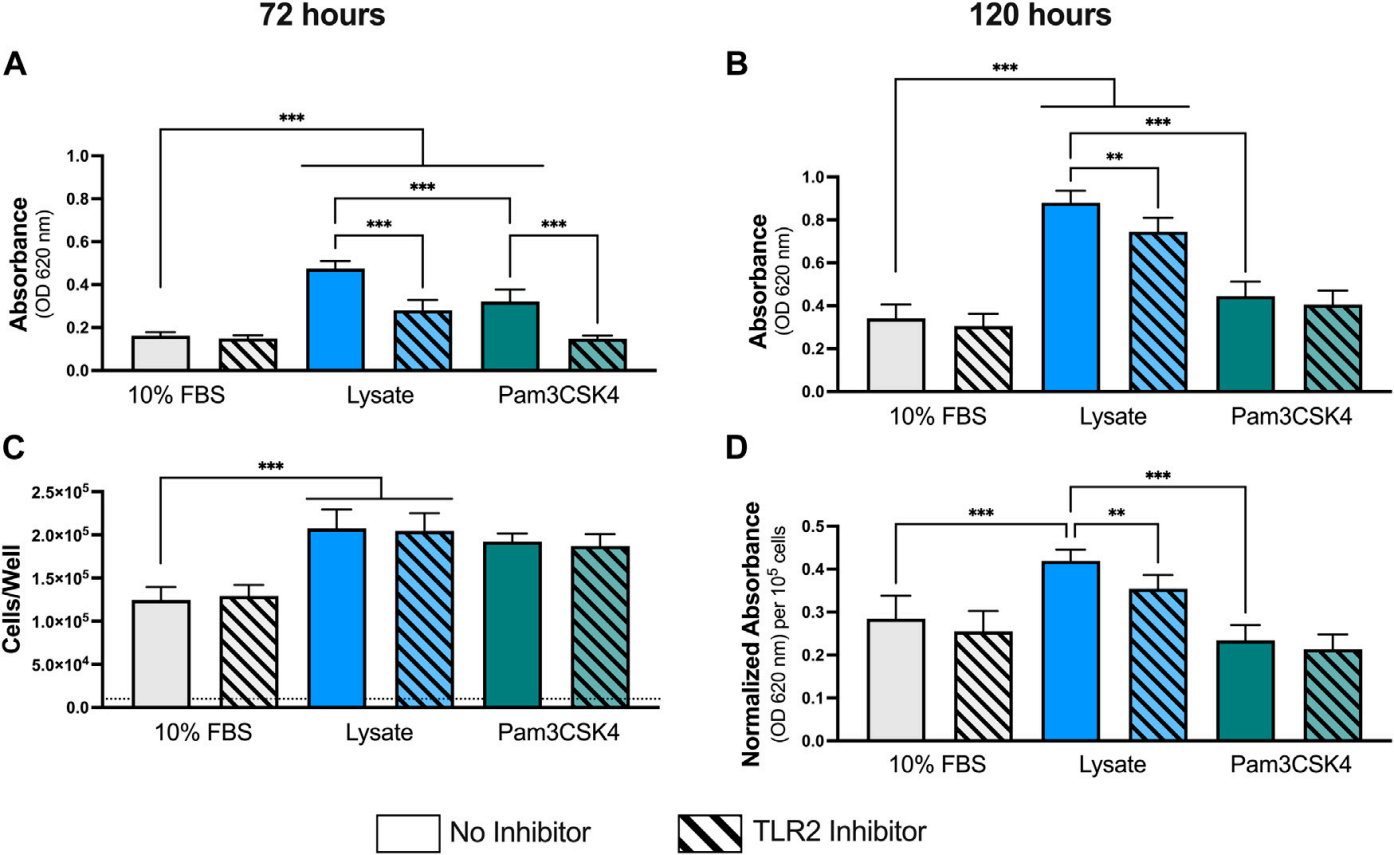
NF-κB/AP-1 activity in RAW-Blue™ macrophages cultured on lysate-conditioned Teflon™ AF surfaces, measured indirectly as absorbance using a SEAP reporter assay. The contribution of TLR2-dependent signaling was investigated by pre-treating cells with a TLR2 neutralizing antibody prior to seeding. Surfaces conditioned with 10% FBS were used as the negative control, and Pam3SCK4 (150 ng/ml) was used as the positive control for TLR2 signaling. The NF-κB/AP-1-dependent SEAP activity (absorbance at 620 nm) in the supernatant was measured at **(A)** 72 h and **(B)** 120 h using a QUANTI-Blue assay. **(C)** The cell number per well at 120 h was determined using a double stranded DNA assay (dotted line indicates seeding density, 1 × 10^4^ cells/well). **(D)** The normalize SEAP activity per cell at 120 h. Data was analyzed using a Brown-Forsythe and Welch ANOVA test with a Dunnet T3 post-hoc test and presented as mean ± standard deviation (*N* = 3, *n* = 9). For clarity, only adjusted p-values (indicated by *p < 0.05, **p < 0.01, ***p < 0.001) for selected comparisons (FBS vs. all conditions, and no inhibitor vs. inhibitor within a stimulant group) are shown in the graphs.

As observed previously at 24 h, macrophages cultured on Teflon™ AF pre-adsorbed with lysate or in the presence of Pam3CSK4 (TLR2 agonist; positive control) had significantly higher NF-κB/AP-1-dependent SEAP activity per well at both 72 h and 120 h of culture, compared to macrophages culture on Teflon™ AF surfaces pre-adsorbed with serum (*p* < 0.05; Figures 1A,B). Lysate-derived adsorbates also yielded higher NF-κB/AP-1-dependent SEAP activity compared to the stimulation with Pam3SCK4 at these extended time points (*p* < 0.001)

Pretreatment with a TLR2 neutralizing antibody had no effect on the macrophage response to the 10% FBS conditioned surfaces at either timepoint (*p* > 0.05 for 10% FBS with and without inhibitor). In contrast, TLR2 inhibition resulted in significant decreases in NF-κB/AP-1-dependent SEAP activity at 72 h and 120 h for macrophages cultured on the lysate-conditioned Teflon™ AF surfaces (*p* < 0.05, compared to the uninhibited Lysate condition). However, the amount of NF-κB/AP-1-dependent SEAP activity following TLR2 inhibition remained significantly higher than the serum-adsorbed condition at both timepoints (*p* < 0.05). In contrast, pre-treatment with the TLR2 neutralizing antibody appeared to attenuate the SEAP activity induced by soluble Pam3SCK4 for both timepoints, yielding similar SEAP activity to the 10% FBS conditions (*p* > 0.05 for both timepoints).

As RAW-Blue™ continue to proliferate following activation, we normalized the SEAP activity to total cell number at 120 h to account for differences in cell numbers among conditions (Figures 1C,D). The number of cells per well at 120 h was measured using a dsDNA QuantiFluor assay (Figure 1C). Adsorbed-lysate and Pam3SCK4 stimulated macrophage cultures had significantly more cells compared with the serum-adsorbed conditions (*p* < 0.05), however there was no significant difference in the number of cells between the adsorbed-lysate and Pam3SCK4 stimulated macrophage cultures (*p* > 0.05). In addition, TLR2 inhibition did not appear to affect the number of cells when compared with their uninhibited counterparts. When the NF-κB/AP-1-dependent SEAP activity was normalized to cell number (Figure 1D), the effect of the lysate-conditioned surface on SEAP activity was maintained (*p* < 0.05 compared to 10% FBS). However, the SEAP activity per cell was similar in the Pam3SCK4 condition compared to the 10% FBS condition.

### 3.2 TLR2-dependent cytokine expression by macrophage cultured on lysate-conditioned Teflon™ AF surfaces

**FIGURE 2:**
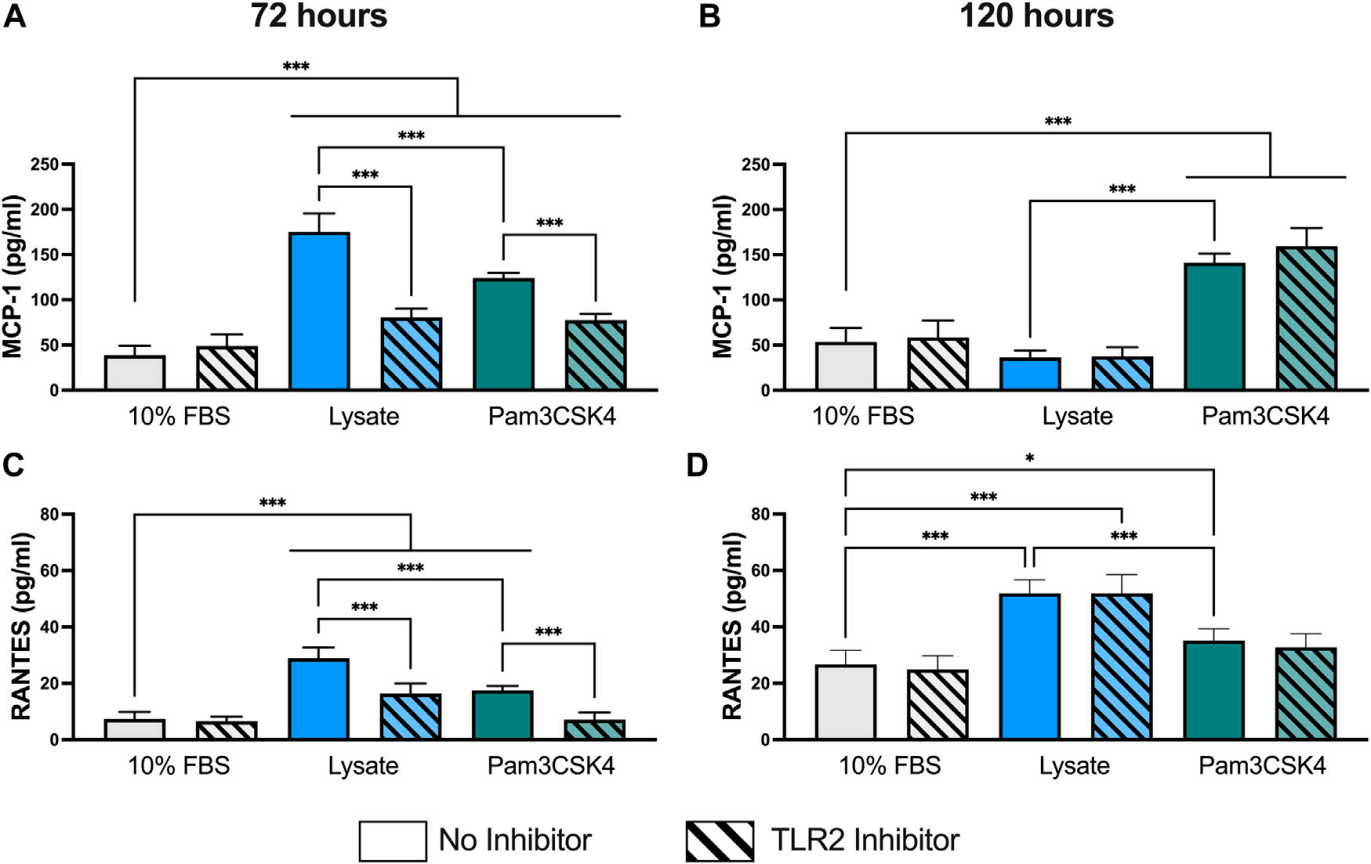
Pro-inflammatory cytokine concentration in the supernatant of RAW-Blue™ cells cultured on serum- or lysate-conditioned Teflon™ AF for 72 h **(A,C)** and 120 h **(B,D)** with or without TLR2 inhibition. The concentrations of MCP-1 **(A,B)** and RANTES **(C,D)** in undiluted supernatant were measured in duplicate using a Luminex multiplexed immunoassay. Data was analyzed using a one-way ANOVA with Tukey’s post-hoc test and presented as mean ± standard deviation (*N* = 3, *n* = 8–9). For clarity, only adjusted *p*-values (indicated by *p < 0.05, **p < 0.01, ***p < 0.001) for selected comparisons (FBS vs. all conditions, and no inhibitor vs. inhibitor within a stimulant group) are shown in the graphs.

Concentrations for MCP-1 and RANTES were successfully measured using the multiplexed Luminex assay in all samples (Figure 2). However, the concentration of IL-1β in undiluted supernatant was below of limit of detection for all samples (data not shown) and the concentrations of IL-6 and IL-10 for most samples were either below the limit of detection or extrapolated below the standard curve (See Supplementary Material for extrapolated and interpolated data for IL-6 and IL-10).

At 72 h, RAW-Blue™ macrophages had higher supernatant concentrations of cytokines MCP-1 (*p* < 0.05) and RANTES (*p* < 0.05) when cultured on lysate-conditioned Teflon™ AF or when treated with Pam3SCK4, compared to serum-adsorbed Teflon™ AF surfaces (Figures 2A,C). Macrophage pre-treatment with the TLR2 neutralizing antibody reduced the concentration of both cytokines in the Lysate and Pam3SCK4 conditions (*p* < 0.05 for MCP-1 and RANTES), relative to their uninhibited counterpart. However, the concentration of both cytokines remained elevated in the TLR2 inhibited Lysate and Pam3SCK4 conditions (*p* < 0.05) compared to the 10% FBS condition.

By 120 h, the concentration of MCP-1 was no longer elevated in the supernatant of lysate-stimulated macrophages (*p* > 0.05 compared to 10% FBS; Figure 2B). However, MCP-1 expression continued to be elevated in response to Pam3SCK4 stimulation (*p* < 0.05, compared to 10% FBS). Notably, the effect of the anti-TLR2 pretreatment was lost by 120 h for all treatment groups. The RANTES concentration in the supernatant of macrophages cultured on lysate-derived adsorbate or treated with soluble Pam3SCK4 treatments continued to be elevated (*p* < 0.05 compared to 10% FBS control; Figure 2D), with lysate-stimulated macrophages producing the highest amount of RANTES per well (*p* < 0.05 compared to 10% FBS and Pam3SCK4). However, the effect of the TLR2 neutralizing antibody was no longer observed at this later timepoint. Combined with the MCP-1 data, these results suggest that either MCP-1 and RANTES secretion was no longer dependent on TLR2 or that the effect of the pre-treatment with the neutralizing antibody had worn off by 120 h.

**FIGURE 3:**
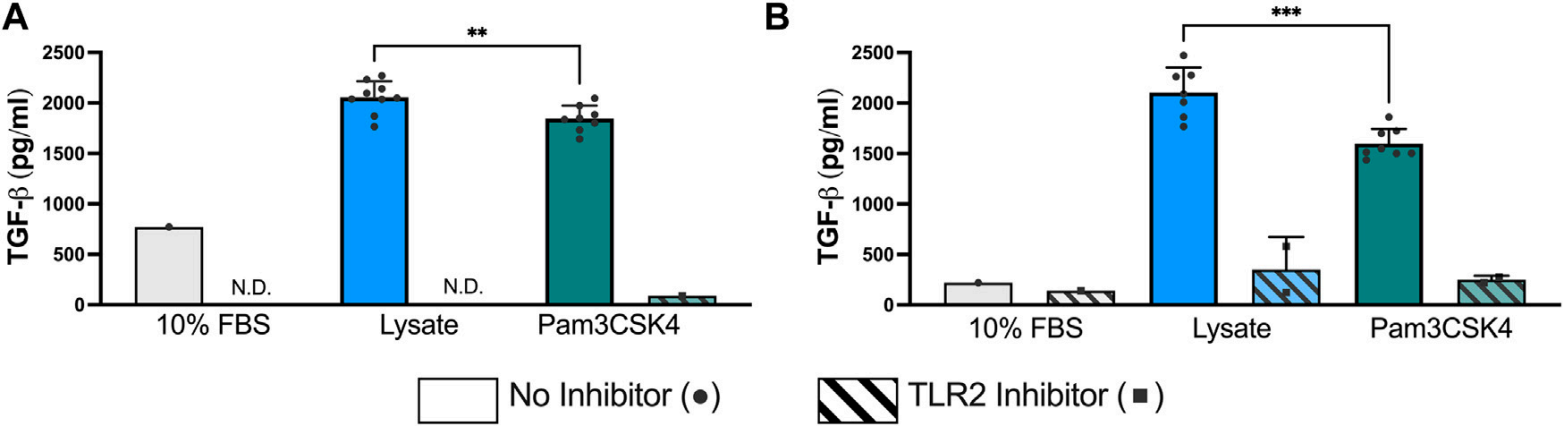
The concentration of TGF-β1 in the supernatant of RAW-Blue™ cells cultured on serum- or lysate-conditioned Teflon™ AF for 72 h **(A)** and 120 h **(B)** with or without TLR2 inhibition. All interpolated data is displayed on the graphs, while missing values were excluded and displayed as not detected (N.D.). Conditions with three or more interpolated concentrations (i.e., Lysate and Pam3SCK4, without inhibitor) were analyzed using an unpaired, two-tailed *t*-test and presented as mean ± standard deviation (*N* = 3, *n* = 9). **p < 0.01, ***p < 0.001.

At 72 h and 120 h, the expression of TGF-β1 was significantly elevated in both the Lysate (2,013 ± 191 pg/ml; 2,070 ± 236 pg/ml, respectively) and Pam3SCK4 groups (1,794 ± 158 pg/ml; 1,557 ± 95 pg/ml, respectively), with lysate-stimulated macrophages producing the highest TGF-β1 concentrations (*p* < 0.05 Lysate vs. Pam3SCK4 at both timepoints) (Figure 3). In comparison, nearly all wells containing macrophages cultured on serum-adsorbed surfaces (with and without inhibitor) had TGF- β1 concentrations that were below range of the standard curve at both time points. For the Lysate and Pam3SCK4 conditions, macrophage pre-treatment with the TLR2 neutralizing antibody resulted in complete attenuation of TGF-β1 expression induced by adsorbed lysate or soluble Pam3SCK4 in seven out of nine wells, at both time points.

### 3.3 Observation of spontaneous macrophage fusion on lysate-conditioned Teflon™ AF surfaces

**FIGURE 4:**
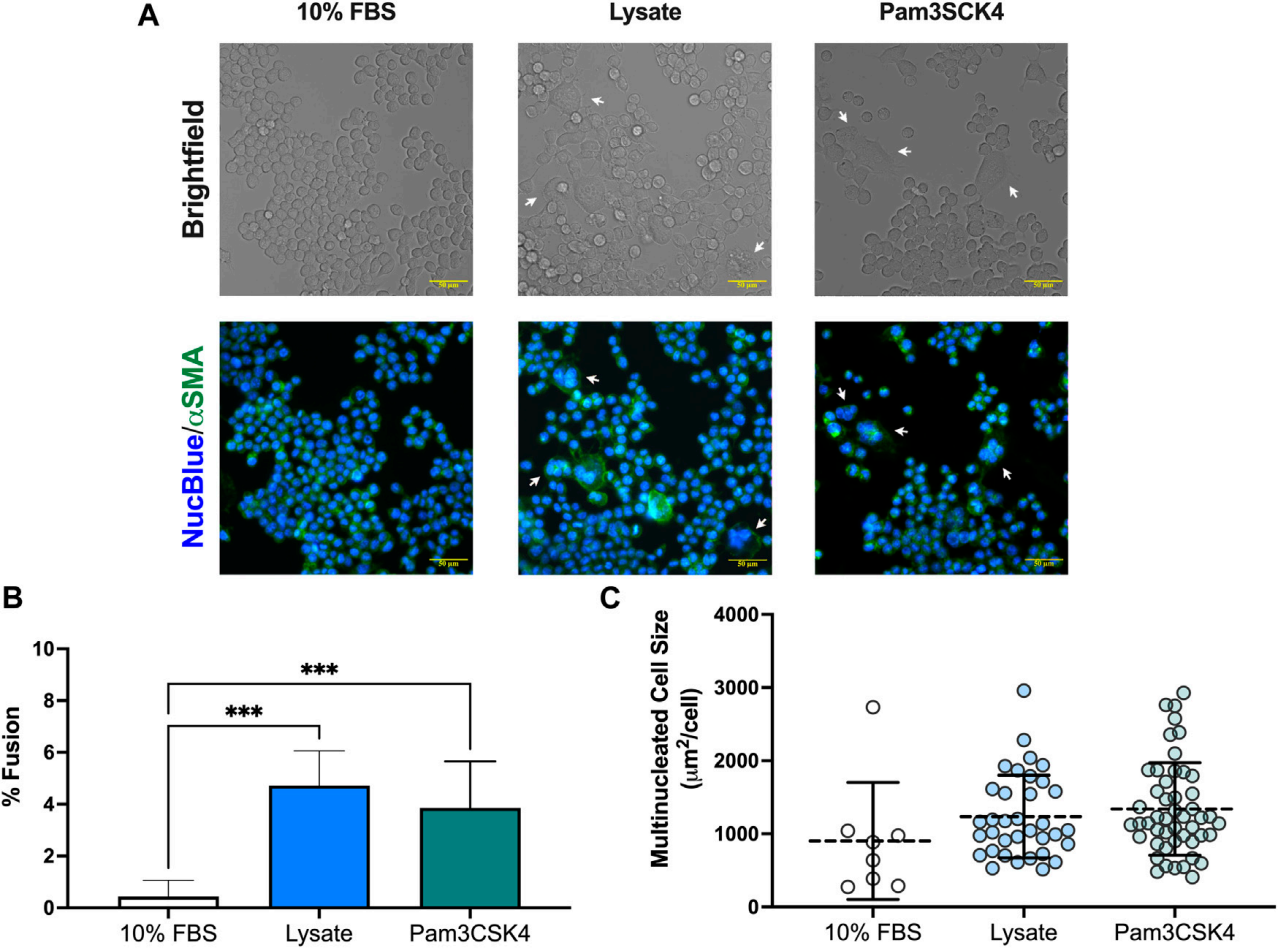
Multinucleated cell formation in RAW-Blue™ macrophages at 72 h. **(A)** Brightfield and fluorescence microscopy images of live mouse RAW-Blue™ macrophages cultured on Teflon™ AF surfaces with adsorbed 10% FBS, absorbed lysate or soluble Pam3SCK4. Cells were stained for nuclei (NucBlue; blue) and plasma membrane (wheat germ agglutinin, green). White arrows highlight multinucleated cells (nuclei > 2). Scare bar: 50 μm. **(B)** Percent fusion of macrophages and **(C)** size of multinucleated cells for three randomly selected fields of view per sample. Data for three independent experiments, with conditions run in triplicate, were analyzed using a one-way ANOVA with Tukey’s post-hoc test and presented as mean ± standard deviation (*N* = 3, *n* = 9, ***p < 0.001).

An unanticipated observation from this study was the presence of multinucleated FBGC in macrophages cultured on lysate conditioned Teflon™ AF surfaces and on Teflon™ AF surfaces with soluble Pam3SCK4 treatment. After 72 h, the presence of FBGC (cells with > 2 nuclei) were observed in wells with lysate-derived adsorbates or Pam3SCK4 (Figure 4A). The macrophage fusion rate was significantly higher for macrophages cultured on adsorbed lysate (4.7% ± 1.3%, *p* < 0.05 vs. 10% FBS) or stimulated with soluble Pam3SCK4 (3.9% ± 1.8%, *p* < 0.05 vs. 10% FBS) than macrophages cultured on serum-derived adsorbates (0.4% ± 0.6%; Figure 4B). There was no statistical difference in the average size of the FBGC among the groups (Figure 4C, *p* > 0.05).

### 3.4 Effect of macrophage conditioned media on fibroblasts

**FIGURE 5:**
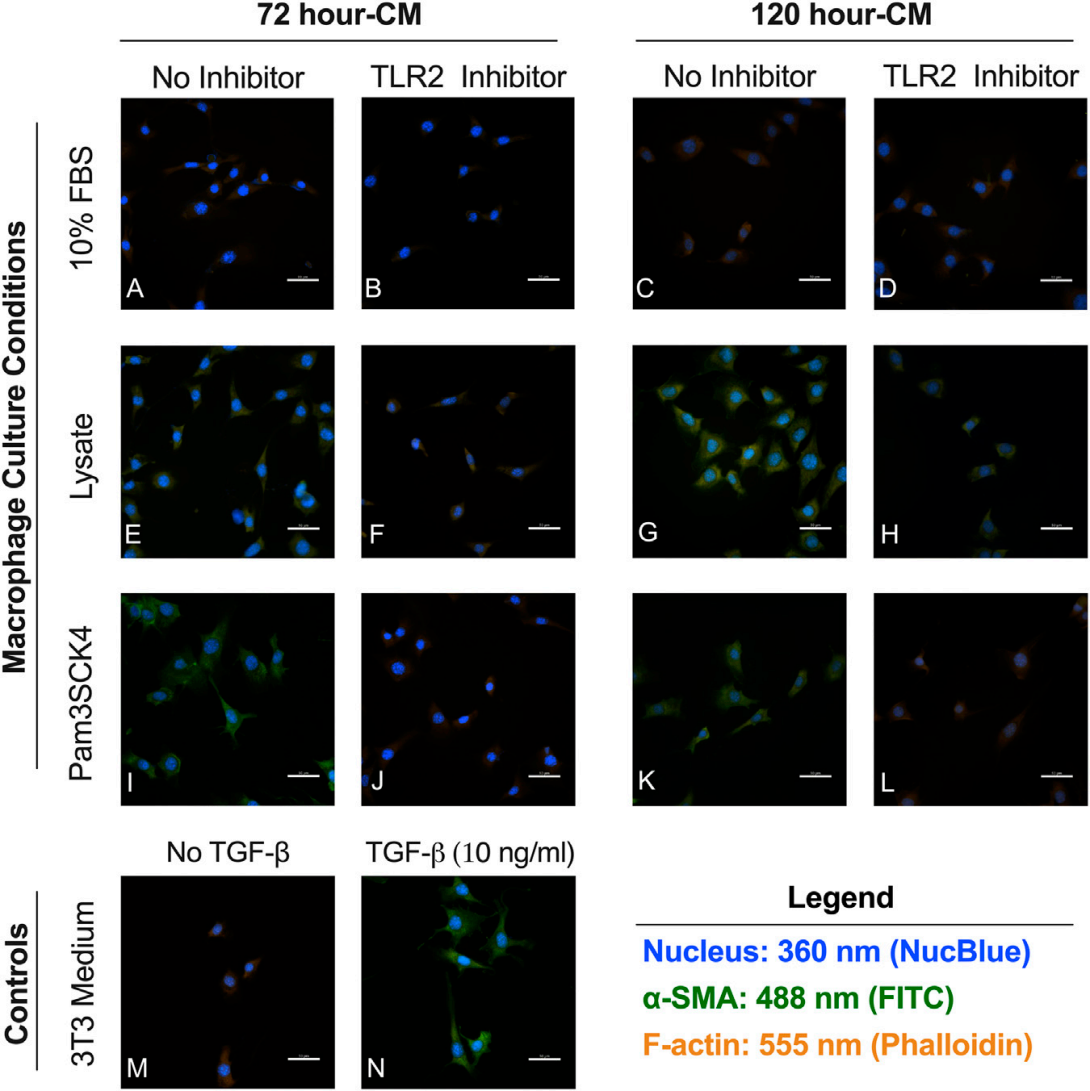
Representative fluorecence microscopy micrographs of fibroblasts cultured for 48 h in conditioned media from RAW 264.7 macrophages cultured on Teflon™ AF pre-conditioned with 10% FBS **(A–D)**, Lysate **(E–H)** or in the presence of Pam3CSK4 **(I–L)**; with or without pre-treatment with TLR2 neutralizing antibodies. Fibroblasts cultured in 3T3 growth medium without **(M)** and with 10 ng/ml TGF-β1 **(N)** were used as the negative and positive fibroblast controls. Cells were stained with an anti-αSMA antibody (green), Hoechst (blue; nucleus), and phalloidin (orange; F-actin). The scale bar is equivalent to 50 µm.

Minimal αSMA staining observed in fibroblasts cultured in the conditioned media from macrophages grown on serum-adsorbed surfaces (with or without TLR2 inhibition) (Figures 5A–D) or in fibroblasts culture in the negative control (unsupplemented 3T3 growth medium, Figure 5M). Fibroblasts cultured in the conditioned media from the Lysate (Figures 5E,G) and Pam3SCK4 (Figures 5I,K) groups stained positive for αSMA, as did fibroblasts stimulated with 10 ng/ml TGF-β1 (positive control; Figure 5N). Modest αSMA staining was also observed for fibroblasts cultured in conditioned media from TLR2 inhibitor Lysate and Pam3SCK4 macrophage groups (Figures 5F,H,J,L). However, the αSMA-positive staining appeared fainter in the fibroblasts grown in conditioned media from Lysate and Pam3SCK4 TLR2 inhibitor groups, compared to their uninhibited counterparts.

**FIGURE 6:**
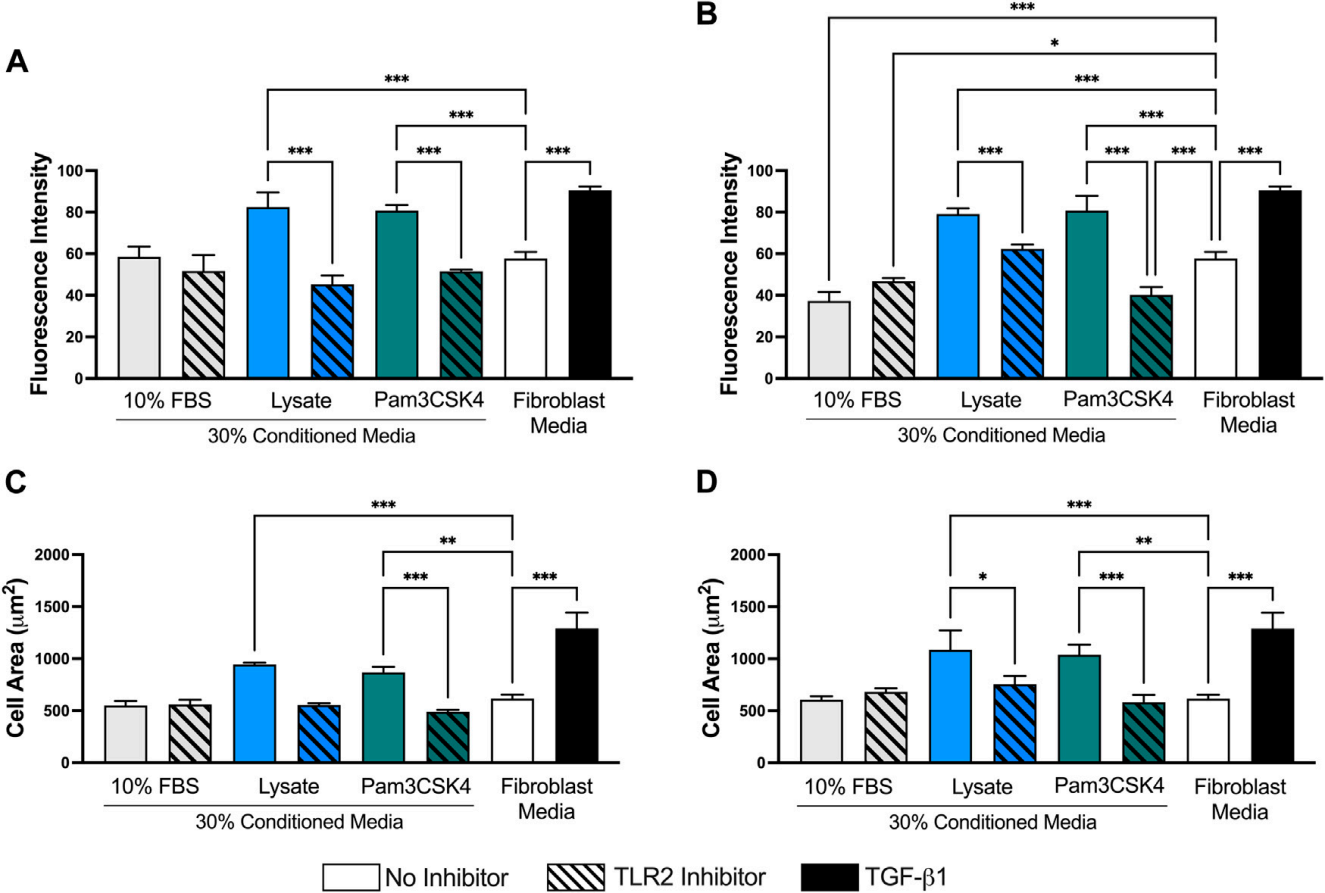
Quantitative analysis performed on 40X micrographs of αSMA (FITC–green), Hoechst (NucBlue–blue), and phalloidin (AlexaFluor 555–orange) stained 3T3 fibroblasts cultured in conditioned media from 72 h **(A,C)** and 120 h **(B,D)** cultures of RAW-264.7 macrophages on serum- or lysate-conditioned Teflon™ AF. The mean fluorescence intensity per cell **(A,B)** was analyzed using the isolated FITC (αSMA) channel while the area of the cell **(C,D)** was determined from the composite images. Three representative images per well were captured at 40x magnification. Microscopy image acquisition settings were kept constant for all conditions. Images were analyzed using ImageJ, and data was presented as mean ± standard deviation (*n* = 3; *p < 0.05).

When the images were analyzed in ImageJ, fibroblasts cultured in Lysate or Pam3CSK4 conditioned media or treated with TGF-β had increased average fluorescence intensity of the FITC (αSMA) channel per cell (*p* < 0.05), compared to fibroblasts cultured in the conditioned media of serum macrophage groups or the negative 3T3 control group (*p* < 0.05, Figure 6). The 72 h and 120 h condition media from Lysate and Pam3SCK4 macrophages groups treated with the TLR2 inhibitor had decreased average fluorescence intensity of αSMA staining, compared to their uninhibited counterparts (*p* < 0.05). Fibroblasts in conditions with significantly higher αSMA expression also had increased size (area per cell), compared to the negative fibroblast control, and this effect was signficantly reduced for groups cultured in conditioned media from TLR2 inhibited macrophage (*p* < 0.05) (Figures 6C,D).

**FIGURE 7:**
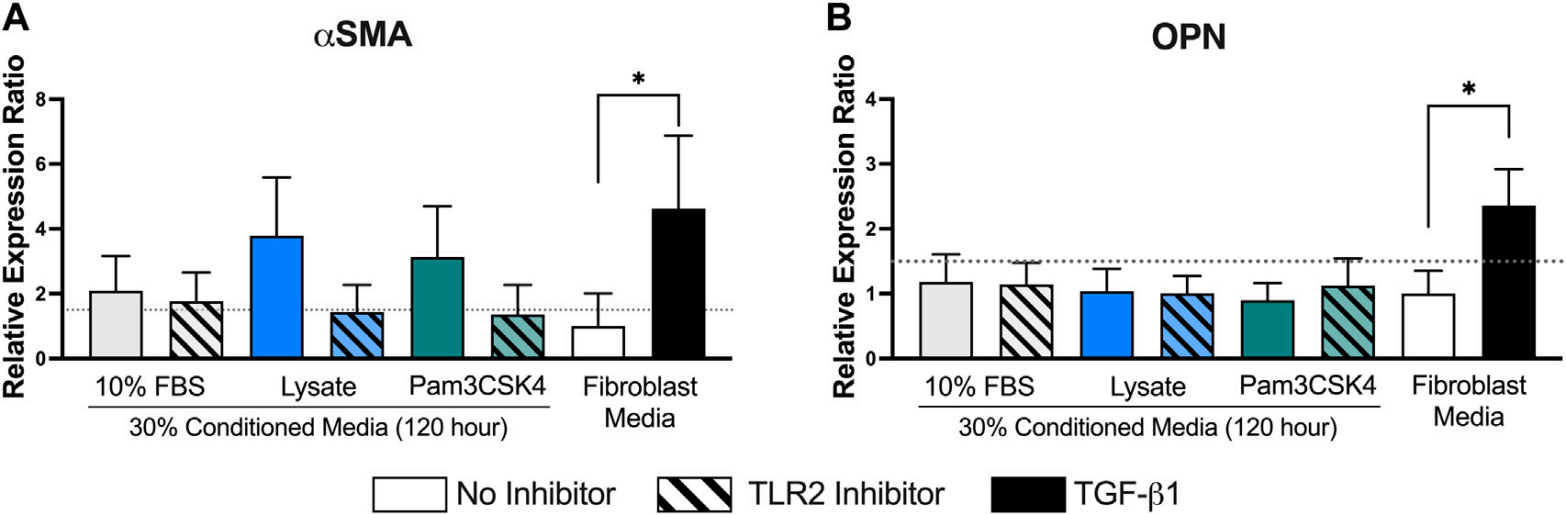
The relative expression ratio of αSMA **(A)** and OPN **(B)** in fibroblasts following 48 h of culture in the conditioned media from RAW 264.7 macrophages cultured under the indicated conditions. mRNA expression was normalized to the negative control (fibroblasts cultured in fibroblast media) and two reference genes (Rplp0 and B2M) using the ddCt method with an efficiency E = 2. Data presented as mean relative expression ± standard error of the ratio (*n* = 3; * indicates 0.67 < *R* > 1.5 and log2NRQ *p* < 0.05), according to Rieu and Powers (2009).

Finally, the relative mRNA expression ratio (R) of two myofibroblast markers, αSMA and OPN, was measured in fibroblasts cultured in the 120-h conditioned macrophage media using qPCR (Figure 7). Trends supporting the fluorescence microscopy αSMA expression analysis were also observed for mRNA expression, where conditioned media from macrophages adhered to lysate-conditioned Teflon™ AF (R = 3.8 ± 1.0 and log_2_NRQ *p* = 0.12 vs. control) or Teflon™ AF with Pam3SCK4 (R = 3.1 ± 0.9 and log_2_NRQ *p* = 0.20 vs. control) increased the fibroblast mRNA expression of αSMA. However, only fibroblasts stimulated with 10 ng/ml of TGF-β1 had a statistically significant increase in αSMA expression (R = 4.6 ± 1.3 and log_2_NRQ *p* < 0.05 compared to negative control; Figure 7A). This condition also induced the expression of OPN (R = 2.4 ± 0.3 and log_2_NRQ *p* < 0.05 compared to negative control; Figure 7B), whereas no change in OPN was observed for the fibroblasts cultured in any of the macrophage conditioned media.

## 4 Discussion

Understanding the various signaling pathways that initiate and guide the progression of host responses to implantable biomaterials is critical to developing effective strategies for modulating adverse implant outcomes related to the FBR. Many signaling pathways, including those of β1 and β2 integrins, mannose receptor and DC-STAMP (Anderson et al., 2008), have been studied extensively in relation to the host response to a range of biomaterials in the context that solely blood-derived adsorbed protein layers mediate the cell-material interface. However, the presence of cell-derived DAMPs within the adsorbed protein layer and their role mediating cell-material interactions through their cognate receptors, such as the TLRs, has not been considered in *in vitro* cell-material interaction models. Previously, our group developed an *in vitro* model of DAMP-adsorption, which used fibroblast lysate as a complex source of intracellular-derived DAMPs [e.g., high mobility group box 1 (HMGB1) and heat shock protein 60 (HSP60)] and studied macrophage activation on surfaces pre-conditioned with lysate (with or without blood-derived proteins) (McKiel and Fitzpatrick, 2018; McKiel et al., 2020). Using this model, we demonstrated that adsorbates derived from fibroblast lysate stimulated a potent TLR2-dependent pro-inflammatory response in the RAW-Blue™ and RAW 264.7 macrophage cell lines, for a range of hydrophobic, non-degradable polymers (e.g., PMMA, PDMS, Teflon™ AF), even when adsorbed in the presence of serum or plasma-derived proteins (McKiel and Fitzpatrick, 2018; McKiel et al., 2020). However, this first study only characterized the acute macrophage response (i.e., 20 h–24 h). In the present study, we examined the proinflammatory and profibrotic signaling in RAW-Blue™ and RAW 264.7 macrophage cell lines in response to Teflon™ AF surfaces pre-adsorbed with lysate at 72 h and 120 h to understand the temporal effect of DAMP-mediated macrophage activation over extended culture periods, and to gain insight into the effect of adsorbed DAMPs on later-stage host response phenomena, specifically the development of a FBR characterized by FBGC formation, chronic inflammation and fibrosis (Anderson et al., 2008). We also examined the extended effect of a single TLR2 neutralizing antibody pre-treatment, administered prior to seeding the macrophages within the various test conditions, to determine if the influence of transient TLR2-neutralization was maintained at later time points or overwhelmed by pro-inflammatory signaling *via* non-TLR2-dependent pathways. Teflon™ AF surfaces pre-conditioned with 10% FBS was used as a reference for macrophage activation in traditional cell-material interaction models, which frequently use FBS or human serum to recapitulate the *in vivo* protein adsorption (Amer et al., 2019; Blatt et al., 2020; Buck et al., 2021).

### 4.1 The effect of lysate-conditioned Teflon™ AF surfaces on macrophage activation

Overall, adherent macrophages on Teflon™ AF surfaces pre-conditioned with 10% FBS displayed a minimal degree of activation over the 120-h culture period, which was unaffected by TLR2 inhibition. These results are consistent with previous work by Grainger and co-workers, in which RAW 264.7 macrophages were cultured on Teflon™ AF surfaces with adsorbates derived from 10% FBS for 21 days (Chamberlain et al., 2015). The RAW 264.7 macrophages had no detectable IL-6 expression and relatively consistent MCP-1 supernatant concentration over the first 7 days, while the RANTES concentration steadily increased from day 1 to day 7 (Chamberlain et al., 2015). These general trends were reproduced in the present study for RAW-Blue macrophages cultured on the 10% FBS-conditioned surfaces. Furthermore, the absence of an effect due to TLR2 inhibition supports established views that macrophage activation in traditional culture environments that use serum-derived adsorbed protein layers does not involve TLR2 and may explain why this signaling pathway has been largely overlooked in sterile biomaterial-induced inflammation and FBR until recently (Li et al., 2019; Liu et al., 2022).

In contrast, lysate-derived adsorbates significantly increased macrophage activation on the Teflon™ AF surfaces at 72 h and 120 h. However, there is a clear transition in the macrophage response between these two time points. At 72 h, surfaces with adsorbed lysate maintained the increased pro-inflammatory activation of adherent macrophages previously observed at 24 h (McKiel and Fitzpatrick, 2018), as indicated by the increased NF-κB and AP-1 transcription factor activity and the increased expression of pro-inflammatory cytokines MCP-1 and RANTES. The lack of detectable IL-6 and IL-1β within the supernatant at these times points suggest the expression of these early phase inflammatory cytokines, which had been present at 24 h (McKiel and Fitzpatrick, 2018), was already resolving by 72 h within this system. The strong expression of TGF-β1 in lysate conditions supports the interpretation that the macrophage response was shifting from an acute/transient inflammatory profile to a chronic/sustained profile, as TGF-β1 is associated with more late-phase responses, including chronic inflammation and tissue formation/fibrotic phases of wound healing and FBR (Moore et al., 2015). At 120 h, macrophages cultured on lysate-conditioned Teflon™ AF surfaces continued to have increased NF-κB/AP-1 activity and RANTES expression when compared to 10% FBS-conditioned surfaces, while MCP-1 expression for the lysate group had returned to levels that appear similar to the 10% FBS control group. This decrease in MCP-1 expression in the lysate group from 72 h to 120 h is consistent with the *in vivo* expression profile of reported for MCP-1 in response to tissue damage (i.e., hind-limb ischemia) and biomaterial implantation models in mice (Shireman et al., 2006; de la Oliva et al., 2018). The RANTES concentrations for all groups increased from 72 h (∼5–35 pg/ml) to 120 h (∼25–55 pg/ml), which reflects the temporal profile described for this cytokine within biomaterial implant sites (de la Oliva et al., 2018). High TGF-β1 expression by macrophages on the lysate-derived adsorbates was maintained at 120 h. Collectively, the macrophage activation profile for lysate-conditioned Teflon™ AF surfaces suggests a transition from a pro-inflammatory state to a pro-fibrotic state by 120 h of culture in response to the adsorbed DAMPs, which reflects aspects of the *in vivo* macrophage response to implanted biomaterials (Luttikhuizen et al., 2007; Rodriguez et al., 2009).

### 4.2 The extended effect the adsorbed lysate compared to a soluble TLR2 agonist

Pam3CSK4 is a synthetic triacylated lipopeptide and is a ligand for the TLR2/1 heterodimer (Jin et al., 2007). In both *in vitro* and *in vivo* models, Pam3CSK4 stimulation has been shown to induce the expression of both pro-inflammatory (e.g., IL-1β, MCP-1, Nos-2) and pro-wound healing (e.g., IL-10, TGF-β) signaling molecules at early timepoints (<24 h) (Funderburg et al., 2011; Stivers et al., 2017). In our model of DAMP adsorption, Pam3CSK4 was used as a positive control for TLR2 signaling and has previously shown similar trends in activation as adsorbed lysate at 24 h (McKiel and Fitzpatrick, 2018; McKiel et al., 2020). In the present study, this TLR2 agonist was applied only once, when the cells were seeded onto the Teflon™ AF surface and would have been diluted upon 50% media changes at day 2 and 4. Therefore, the Pam3CSK4 condition was considered to model an extended macrophage response to an acute, individual TLR2 ligand, compared to the persistent TLR2 agonists within the adsorbed lysate layer. It is important to note that the comparisons between lysate and Pam3CSK4 is intended to focus on the general trends, and not the relative degree of macrophage activation because a higher concentration of Pam3CSK4 would elicit a stronger response (Staquet et al., 2011).

At 72 h, macrophages cultured on lysate-derived adsorbates yielded a similar overall trend in macrophage activation as the single 150 ng/ml treatment of Pam3CSK4, with both conditions having elevated transcription factor activity and expression of MCP-1, RANTES and TGF-β1 compared to the 10% FBS control. Both treatments stimulated macrophage fusion at 72 h on TCPS surfaces, and the conditioned media from both treatment groups appeared to induce a similar response from fibroblasts. However, differences between the Lysate and Pam3SCK4 conditions appeared at 120 h. While macrophages cultured on lysate-conditioned surfaces continued to have increased NF-κB/AP-1 activity at 120 h compared to the control, no significant differences were observed for the Pam3SCK4 stimulated macrophages. The expression of MCP-1 was also significantly different between the Lysate and Pam3SCK4 conditions, where MCP-1 was significantly increased by Pam3SCK4 treatment compared to both Lysate and 10% FBS. No IL-1β or IL-10 was detected for the Pam3CSK4-stimulated macrophages at 72 h or 120 h, despite reports of Pam3CSK4 inducing their expression at earlier timepoints (Funderburg et al., 2011; Stivers et al., 2017). The differences in the NF-κB/AP-1 activity and MCP-1 concentrations between Pam3CSK4 and Lysate groups likely reflect the difference between an individual TLR2-specific agonist and lysate, which is a complex mixture of proteins, as well as the method of presentation (i.e., soluble vs. adsorbed).

### 4.3 The effect of TLR2 neutralizing antibody pre-treatment

The single TLR2 neutralizing antibody pre-treatment had a significant effect on the macrophage response to lysate-conditioned surfaces at 72 h, with TLR2 inhibition decreasing both the activity of NF-κB/AP-1 and cytokine concentration of MCP-1, RANTES and TGF-β1 in the conditioned media. No effect of TLR2 neutralization was observed for macrophages cultured on serum-derived adsorbates. These results show that acute TLR2 inhibition of NF-κB and AP-1 transcription factor activity impacted both proinflammatory signaling (i.e., decreased MCP-1 and RANTES) and profibrotic signaling (i.e., decreased TGF-β1) in response to adsorbed-lysate on Teflon™ AF at 72 h. This is consistent with the roles of NF-κB and AP-1, as they are known to regulate the expression of various proinflammatory cytokines, including MCP-1 and RANTES (Rovin et al., 1995; Kim et al., 2006; Liu et al., 2017). TGF-β1 expression is regulated, in part, by the AP-1 family and also has NF-κB binding sites in TGFB1 promoter region (Birchenall-Roberts et al., 1990; Zhou et al., 2020). A significant observation in this extended study was the TLR2-dependent induction of TGF-β1 expression, which was abolished by TLR2 inhibition. By 120 h, the neutralizing antibody pre-treatment maintained a slight but significant reduction in the NF-κB/AP-1-dependent SEAP activity and had no effect on the MCP-1 and RANTES concentration, suggesting that the transient effect of the TLR2 antibody on the pro-inflammatory signaling was coming to end. However, the anti-TLR2 treated macrophages continued to have almost complete attenuation of the lysate-induced TGF-β1 expression. The same overall trends for TLR2 inhibition were observed for the Pam3CSK4-treated macrophages. Previously, it was shown that TLR2 knockout mice generated smaller fibrotic scars in response to angiotensin II-induced cardiac fibrosis, which was attributed to the decreased TGF-β1 at the wound site (Wang et al., 2014). Similarly, TLR2 neutralizing antibodies effectively blocked TGF-β1 production in primary mouse bone marrow-derived macrophages in response to Pam3SCK4 (Foley et al., 2012).

There are multiple scenarios that may explain the loss of effect in response to the TLR2 inhibition provided by the pre-treatment of the neutralizing antibody. It may reflect the transient inhibition that is achieved by neutralizing antibodies, due to the turn-over of receptor molecules at the cell surface. It is also possible that the gradual exposure to the adsorbed DAMPs (instead of a sudden exposure of naïve cells to high numbers of ligand) generate a more muted response. A second explanation is that blocking the acute inflammatory response lowered the overall expression of paracrine pro-inflammatory factors (e.g., cytokines, chemokines, eicosanoids etc.) and stunted the amplification of the inflammatory response, resulting in less macrophage activation and a dampened response to the adsorbed lysate molecules. Future studies using sustained delivery or repeated treatments using a TLR2 pathway inhibitor, or the use of TLR2-knockout macrophages would be expected to address the question of whether the loss of the effect of TLR2 inhibition in the present study was due to the transient nature of the receptor neutralization or because the overall macrophage response was no longer strongly influenced by TLR2.

### 4.4 Spontaneous macrophage fusion in response to TLR2 agonists

By 72 h, the macrophages cultured on lysate-conditioned surfaces and in the Pam3CSK4 treatment group both had noticeable formation of FBGCs, despite the absence of known exogenous fusogenic stimuli, such as IL-4, IL-13, receptor activator of nuclear factor κ-B ligand (RANK-L), or α-tocopherol (Pereira et al., 2018). To quantify the frequency of macrophage fusion in lysate-conditioned surfaces, macrophages were cultured of lysate-conditioned TCPS for 72 h using higher seeding densities to facilitate fusion. The degree of fusion observed in the adsorbed lysate and Pam3CSK4 conditions were significantly higher than fusion on serum adsorbates, suggesting the TLR2 stimulation created a fusion permissive environment for the macrophages, either by inducing the production fusogenic cytokines IL-4 and IL-13 and/or other molecules that support macrophage fusion. While macrophages are generally not considered to be sources of Th2 cytokines IL4 and IL-13 (Junttila, 2018), previous *in situ* staining of FBR microenvironments in mice showed co-localization of IL-4 and IL-13 with F4/80+ cells in areas of FBGC and macrophage fusion (Higgins et al., 2009). There is also evidence that MCP-1 participates in macrophage fusion (Kyriakides et al., 2004), so the high concentrations of MCP-1 observed at 72 h for the lysate and Pam3CSK4 stimulated macrophages likely contributed to the early occurrences of macrophage fusion. However, further studies are required to determine the molecular mechanism of the incidental fusion observed here. It is important to note that the percent fusion (∼5%) and size of the multinucleated cells reported here are much lower than those reported in studies that use exogenous fusion factors, such as IL-4 or IL-13 (Jones et al., 2007; McNally and Anderson, 2011, 2015). However, these studies employ optimized conditions to support macrophage fusion, in contrast to the spontaneous fusion observed on the DAMP-adsorbed surfaces (DeFife et al., 1997; McNally et al., 2008; Pereira et al., 2018; ten Harkel et al., 2015). Spontaneous macrophage fusion (i.e., in the absence of exogenous fusion factors) for primary mouse and human macrophages has been reported for various surface chemistries and topographies (Jones et al., 2007; Moon et al., 2016). Although the rate of spontaneous fusion was similarly low, macrophage fusion in these other systems was not observed until later culture times (e.g., 5–7 days) (Jones et al., 2007; Moon et al., 2016). Our preliminary observation of spontaneous macrophage fusion in response to TLR2 agonists require further investigation to determine molecular mechanisms as well as optimize the experimental condition to better study this phenomenon.

### 4.5 Pro-fibrotic signaling in TLR2-stimulated macrophages

The indirect co-culture model using macrophage conditioned media in fibroblast cultures demonstrated a functional, downstream impact of TLR2-dependent signaling in macrophages. Fibroblasts cultured for 48 h with conditioned media from adsorbed lysate and Pam3CSK4 stimulated macrophages had increased αSMA protein expression and cell spreading (indicated as cm^2^/cell), indicative of myofibroblast differentiation (Thannickal et al., 2003; Sousa et al., 2007; Shinde et al., 2017). TLR2 inhibition of the macrophages significantly decreased the amount of fibroblast αSMA expression and cell spreading in both the Lysate and Pam3CSK4 groups, indicating that TLR2 inhibition attenuated the overall expression of profibrotic factors by lysate (or Pam3CSK4) stimulated macrophages, including TGF-β1 (Baum and Duffy, 2011). The qPCR results support this conclusion, as a trend of increased αSMA expression was observed in response to Lysate and Pam3CSK4 conditioned media, in comparison with the conditioned media from macrophages treated with the TLR2 neutralizing antibody. However, the lack of increased osteopontin expression among fibroblast groups exposed to any of the macrophage conditioned media suggests that the conditioned media did not induce as robust a differentiation towards a myofibroblast phenotype as the exogenous TGF-β1 control. This is likely due to the lower amount of TGF-β1 in the adsorbed-lysate and Pam3CSK4 stimulated macrophages conditioned media (approximately 0.6 ng/ml) compared to the exogenous TGF-β1 (10 ng/ml). Chen and Thibealt previously demonstrated that 0.1 ng/ml did not induce myofibroblast differentiation after 72 h, while 1 ng/ml did (Chen and Thibeault, 2012). Several studies have also examined myofibroblast differentiation in co-cultures of macrophages and fibroblasts *in vitro* and found similar results to those presented in the present study. Myofibroblast differentiation typically began 72 h after co-culturing with M1 polarized macrophages and between 24 and 48 h following co-culture with M2 polarized macrophages (Zhu et al., 2017; Hou et al., 2018; Li and Bratlie, 2021; Sapudom et al., 2021). Consequently, it is likely that combined effect of TGF-β1 and other cytokines in the conditioned media was sufficient to inducing αSMA production in the fibroblast cultures at the 48-h time point used here, and it is likely that a mature myofibroblast phenotype would be achieved at later time points and/or using a direct co-culture system.

### 4.6 Study limitations

The present study used the RAW-Blue macrophage cell line to enable the rapid and quantitative indirect measurement of NF-κB/AP-1 activity and the interrogation of TLR2 neutralization on NF-κB/AP-1 signaling. Although the RAW-Blue line and the RAW 264.7 parental strain are commonly used macrophage models, it is well established that macrophage cell lines have limitations in modelling primary macrophage responses, as well as species-specific differences in TLR signaling (Chamberlain et al., 2009; Sun et al., 2016; Das et al., 2018). Therefore, follow-up studies using primary macrophages and *in vivo* models exploring the impact of TLR2 in the host response to IIS cannulas is required.

A further limitation of the current study is the uncharacterized composition of the adsorbates derived from fibroblast lysate, and how it compares to those derived either from serum *in vitro* or acquired *in vivo.* Thus far, we have attributed the macrophage response on lysate-adsorbed surfaces to the presence of DAMPs within the adsorbed layer based on the source of the molecules (i.e., cell-derived lysates) and the ability of the adsorbed lysate to activate pattern recognition receptors, such as TLRs. However, proteomic analysis of the lysate-derived proteins layers is required to address this model limitation and confirm the presence of known DAMPs within the adsorbates and their ability to adsorb in competition with blood proteins. In terms of characterizing the composition of *in vivo* adsorbed protein layers, a proteomics analysis of adsorbed proteins was reported for polyethylene glycol (PEG) hydrogels following a 30-min implant within a dorsal subcutaneous pocket in male mice (Swartzlander et al., 2015). A number of well-known DAMPs, including HMGB1, heat shock proteins and peroxiredoxins, were identified on the hydrogel surfaces [please refer to Supplementary Table S1 in Swartzlander et al. (2015)], providing evidence that DAMPs are present within adsorbed protein layers *in vivo*. However, the composition of adsorbed protein layers for a variety of materials and implant locations should be characterized to obtain a more complete understanding of the true diversity of adsorbed protein layers formed *in vivo* and to determine if current protein adsorption and cell-material interaction models can be improved upon.

### 4.7 Emerging evidence that TLR2 mediates biomaterial host responses

The present work adds to the growing evidence of the importance of TLR2 in the host response to biomaterials. As described earlier, our group has shown using *in vitro* models of DAMP adsorption that TLR2 inhibition decreased NF-κB and AP-1 transcription factor activity and the expression of IL-6 and TNF-α in RAW-Blue™ and RAW 264.7 macrophages on lysate-conditioned TCPS, PMMA, PDMS, and Teflon™ AF (McKiel and Fitzpatrick, 2018; McKiel et al., 2020). The results presented here support the hypothesis that TLR2 plays a significant role in the development of the FBR and that the TLR2 pathway represents a potential target for modulating both the acute and chronic stages of the host response. Others have shown that IL-4 stimulated human monocyte-derived macrophages express TLR2 after 7 days on multiple materials, and that the expression level was dependent on material surface properties (McNally and Anderson, 2015). In contrast, TLR4 and TLR5 expressions were not detectable at 7 days, indicating TLR2 played a more prominent role in the response of the macrophages to the material surface (McNally and Anderson, 2015). In addition, myeloid differentiation primary response 88 (MyD88), an adaptor protein for many TLRs including TLR2, has been shown to be involved in the inflammation and fibrosis resulting from polyethylene glycol (PEG) hydrogel implants (Amer et al., 2019). MyD88-deficient mice had reduced inflammatory cell recruitment and thinner fibrous capsules when compared to wild type mice, 28 days after PEG hydrogel implantation (Amer et al., 2019). In a recent study by Liu and colleagues identified the TLR2-encoding gene *Tlr2* as a critical gene hub in the FBR to silicone implants in rats (Liu et al., 2022). While collectively these studies provide evidence that TLR2 is a promising candidate as a therapeutic target for modulating biomaterial host responses, further studies in primary mouse and human macrophages and *in vivo* studies focusing on TLR2 inhibition are needed to determine the relative importance of this pathway in the context of the complex *in vivo* response for a broad range of biomaterials.

## Data Availability

The raw data supporting the conclusion of this article will be made available by the authors, without undue reservation.
