# Supplementary material for: The extended effect of adsorbed damage-associated molecular patterns and Toll-like receptor 2 signaling on macrophage-material interactions
Source: Front Bioeng Biotechnol. 2022 Aug 26;10:959512. doi: 10.3389/fbioe.2022.959512 (PMC9458975; doi:10.3389/fbioe.2022.959512)
Supplement: Supplementary file 1 [file Image1.pdf]

Supplementary Material

1.1 Supplementary Figures

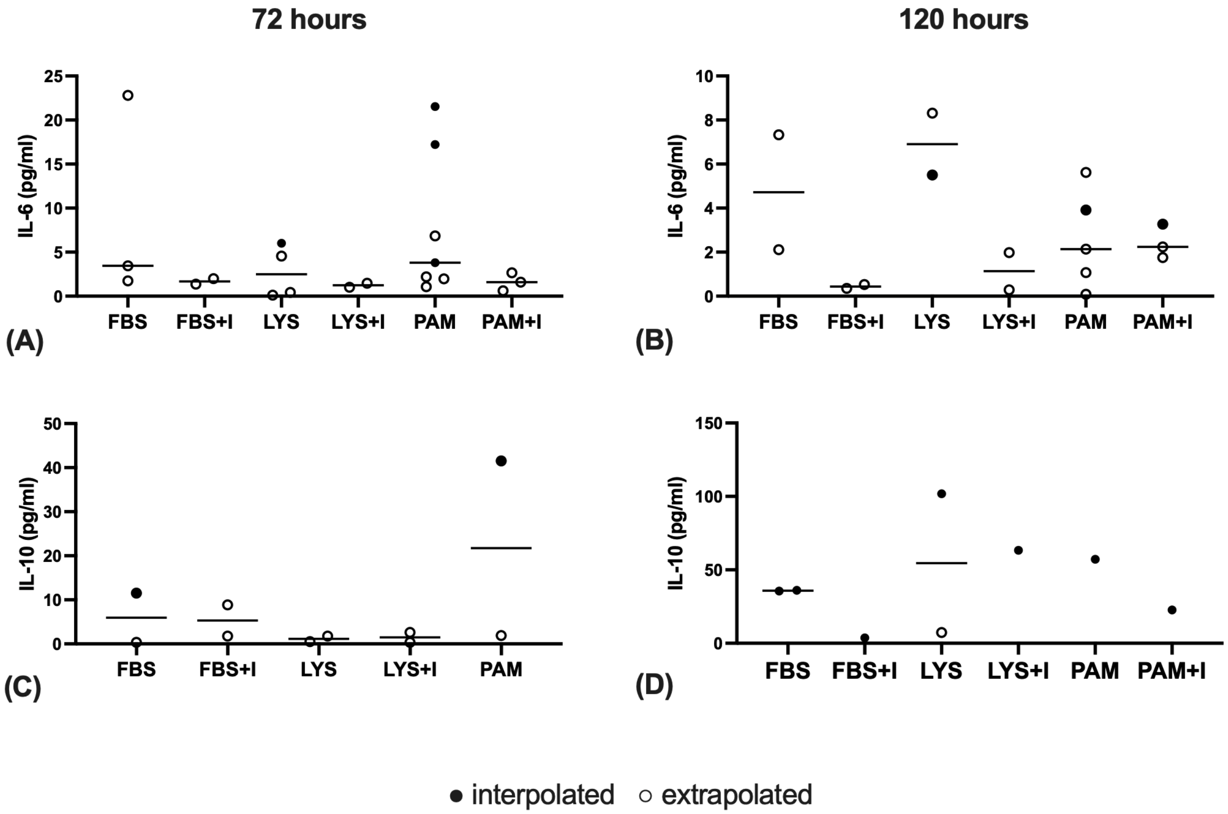

Supplementary Figure 1. Concentration of IL-6 and IL-10 in supernatant of RAW-Blue™ cells cultured on serum-adsorbed or lysate-adsorbed Teflon™ AF for 72 (A, C) and 120 hours (B, D) with or without TLR2 inhibition. The concentrations of IL-6 (A, B) and IL-10 (C, D) were measured in duplicate using undiluted supernatant using a Luminex multiplexed immunoassay (n = 9). The concentrations for samples with absorbances above the limit of detection were interpolated or extrapolated from the standard curve.
